# Cancer Screening by Systemic Administration of a Gene Delivery Vector Encoding Tumor-Selective Secretable Biomarker Expression

**DOI:** 10.1371/journal.pone.0019530

**Published:** 2011-05-11

**Authors:** Andrew W. Browne, Jennifer L. Leddon, Mark A. Currier, Jon P. Williams, Jason S. Frischer, Margaret H. Collins, Chong H. Ahn, Timothy P. Cripe

**Affiliations:** 1 Division of Oncology, Cincinnati Children's Hospital Medical Center, Cincinnati, Ohio, United States of America; 2 Division of Experimental Hematology, Cincinnati Children's Hospital Medical Center, Cincinnati, Ohio, United States of America; 3 Division of Surgery, Cincinnati Children's Hospital Medical Center, Cincinnati, Ohio, United States of America; 4 Division of Pathology, Cincinnati Children's Hospital Medical Center, Cincinnati, Ohio, United States of America; 5 Physician Scientist Training Program, University of Cincinnati College of Medicine, Cincinnati, Ohio, United States of America; 6 Department of Electrical and Computer Engineering, University of Cincinnati, Cincinnati, Ohio, United States of America; Health Canada, Canada

## Abstract

Cancer biomarkers facilitate screening and early detection but are known for only a few cancer types. We demonstrated the principle of inducing tumors to secrete a serum biomarker using a systemically administered gene delivery vector that targets tumors for selective expression of an engineered cassette. We exploited tumor-selective replication of a conditionally replicative Herpes simplex virus (HSV) combined with a replication-dependent late viral promoter to achieve tumor-selective biomarker expression as an example gene delivery vector. Virus replication, cytotoxicity and biomarker production were low in quiescent normal human foreskin keratinocytes and high in cancer cells *in vitro*. Following intravenous injection of virus >90% of tumor-bearing mice exhibited higher levels of biomarker than non-tumor-bearing mice and upon necropsy, we detected virus exclusively in tumors. Our strategy of forcing tumors to secrete a serum biomarker could be useful for cancer screening in high-risk patients, and possibly for monitoring response to therapy. In addition, because oncolytic vectors for tumor specific gene delivery are cytotoxic, they may supplement our screening strategy as a “theragnostic” agent. The cancer screening approach presented in this work introduces a paradigm shift in the utility of gene delivery which we foresee being improved by alternative vectors targeting gene delivery and expression to tumors. Refining this approach will usher a new era for clinical cancer screening that may be implemented in the developed and undeveloped world.

## Introduction

**Figure 1:**
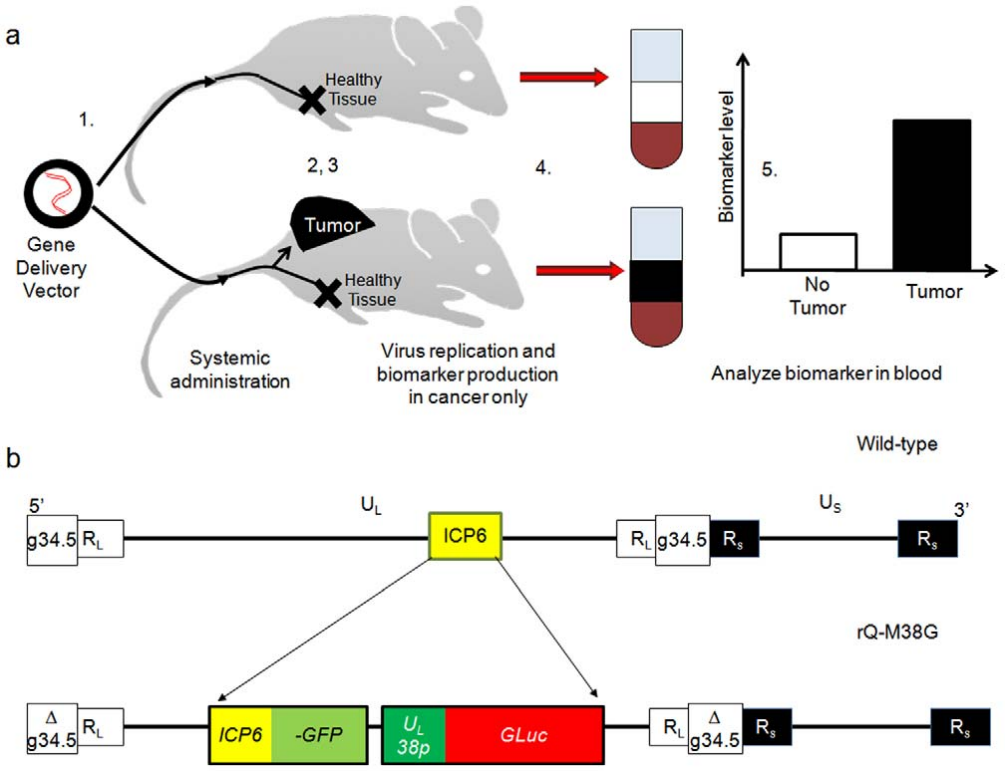
Overall strategy for exogenous cancer biomarkers. (**a**) Steps for cancer screening strategy are as follows: 1) Cancer-targeting Herpes Simplex Virus (HSV) is injected systemically. 2) Engineered HSV selectively replicates in tumors while being cleared from healthy non-cancerous tissues. 3) Biomarker is selectively produced in tumors. 4) Blood samples are collected and analyzed for biomarker levels. 5) Serum levels of exogenously delivered biomarker are higher in tumor bearing mice than healthy tumor-free mice. (**b**) Gene maps for wild-type HSV and novel recombinant rQ-M38G.

Early cancer detection is vital to improve cure rates because cancer stage predicts prognosis. Cancer-associated blood biomarkers have been identified in a few cancers such as prostate specific antigen (PSA) in prostate cancer and alpha fetoprotein in some liver and germline cancers. Biomarkers have not been identified for most pediatric cancers and many adult cancers. New innovations in systemic gene transfer raise the prospect of selectively delivering and activating genes encoding easily detectable biomarkers into tumor cells that do not produce known serum biomarkers. We sought to develop a prototypical cancer screening strategy whereby genetic information encoding a universal serum biomarker for cancer would be injected into a patient systemically, delivered to and expressed within tumor cells in a tumor-selective manner. Tumors would effectively be forced to secrete a serum biomarker, which could then be measured in the blood or urine as a screening test while tumor-free patients would show no or only low levels of biomarker following systemic administration of the gene delivery vector (Fig. 1a).

Exogenously administered gene-encoded biomarkers require tumor-targeted gene delivery and/or expression. Targeting small molecules and particles to tumors can be achieved by passive Enhanced Permeability and Retention (EPR) [1], [2], [3], ligand-guided active targeting [4], [5], [6], [7], tumor microenvironment-dependent targeting [8], [9], [10], or a combination of each [11]. Viruses are naturally occurring nanoparticles optimized to deliver genetic information into target cells. The major advantage of viruses over non-viral vectors for DNA delivery is their inherent predilection for replication in tumors and consequent amplification of signal.

The Herpes Simplex Virus (HSV) type 1 is a model gene delivery vector because it can infect a wide range of human cell types, transduce both dividing and quiescent cells efficiently, be engineered to express transgene products, accept transgenes driven by heterologous or homologous promoters, is epigenomic, has scalable production, and can safely be controlled with antiviral drugs [12]. Selective mutations in HSV genes confer cancer-selective viral replication. Mutation in the HSV genes encoding the ribonucleotide reductase large subunit (ICP6/U_L_39) and the late viral protein ICP34.5 (γ_1_34.5) limits robust viral replication to tumors [13], [14], [15], [16] and have been shown to be safe in clinical trials. Activation of the strict late viral U_L_38 gene is dependent upon preceding sequential activation of immediate early and early viral genes and cellular transcription factors [17] and the U_L_38 promoter (U_L_38p) has been demonstrated to be selectively activated in cancer cells in the context of replication competent γ_1_34.5^−/−^ HSV mutants [18]. The dependence of late gene expression upon activation by early genes makes U_L_38p a strong candidate for delivery of transgenes to cancer cells with selective expression in the context of a double mutant HSV lacking ICP6 and γ_1_34.5.

We developed an HSV as an exemplar gene delivery vector for inducing biomarker secretion selectively from tumors. Others have incorporated genes encoding secretable biomarkers to oncolytic viruses as reporters for virus activity [19], [20], [21], [22] and gene cassettes for non-invasive monitoring viral delivered genes [23]. Sodium-iodide symporter genes encoded in oncolytic viruses also facilitate nuclear medicine imaging and treatment in infected tumors [24], [25]. Recently, Gaussia luciferase (GLuc) was identified and developed as a powerful new reporter molecule [26] that is readily secreted from cells making it useful for both *in vitro* and *in vivo* applications where expression kinetics are of interest. We employed GLuc as a sample biomarker for this proof of principle because GLuc is 1000 times brighter than other luciferases, is more sensitive than secretable alkaline phosphatase, and is detectable in blood and urine *in vivo* [27], [28]. Using a directed recombination approach [29], we engineered an HSV mutant, rQ-M38G, with GLuc under the control of the late viral promoter U_L_38 (Fig. 1b).

We sought to evaluate our engineered viral gene delivery vector in animal models for several different tumor types formed different locations: intraperitoneal, subcutaneous, intramuscular and orthotopic intrarenal tumors. Following systemic injection of our gene delivery vector (rQ-M38G) into the blood of tumor-bearing mice, we observed rQ-M38G producing cancer cell-dependent cytotoxicity and biomarker production. We also noted several instances where rQ-M38G was able to force microscopic tumor burdens to produce detectable blood biomarker. These experimental trials demonstrated the principle of detecting tumors by forcing them to express a secretable biomarker.

## Results

### *In vitro* characterization of mutant HSV rQ-M38G

**Figure 2:**
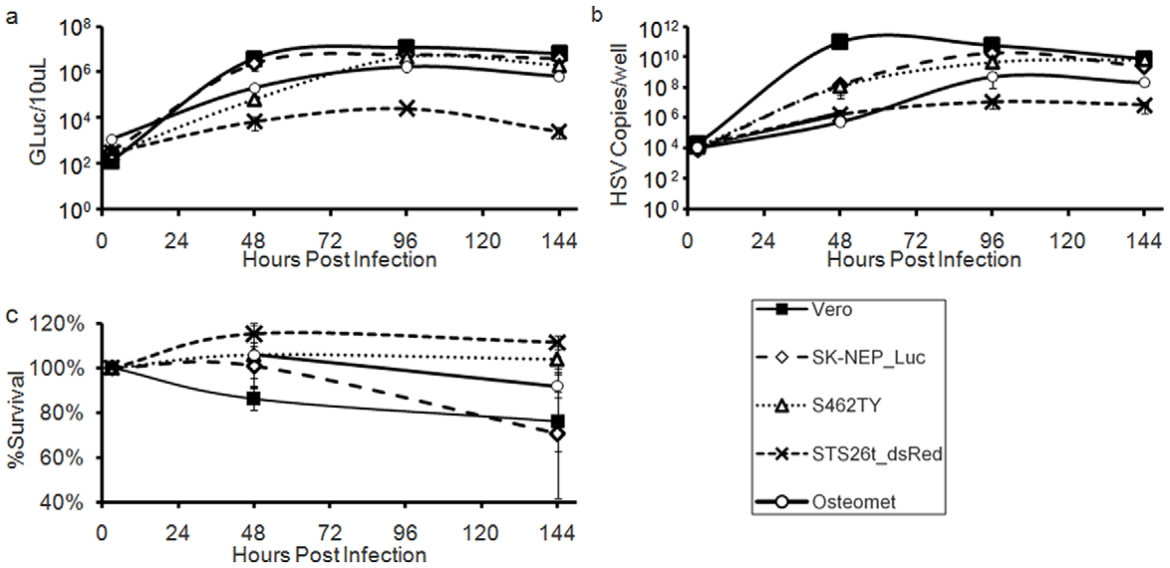
Differential sensitivity of cell lines to virus infection. (**a**) Gaussia luciferase (GLuc) transgene expression, (**b**) virus replication, and (**c**) cytotoxicity following infection of Vero cells and a panel of human tumor cell lines with rQ-M38G (MOI = 0.001).

rQ-M38G-mediated GLuc transduction, replication and cytotoxicity were tested by infecting a range of cell types with various virus concentrations and assessing GLuc levels in culture media (Fig. 2a, Fig. S1), virus genome copy number (Fig. 2b, Fig. S2) and cytotoxicity (Fig. 2c, Fig. S3) on days 2, 4, and 6 after infection. Cell replication-dependent cytotoxicity was observed in replicating human foreskin keratinocytes (HFK-r) while cytotoxicity was attenuated or absent in differentiated/quiescent human foreskin keratinocytes (HFK-q) (Fig. S3). Vero, an African green monkey kidney cell line that is known for permissive HSV replication, showed high GLuc expression and rQ-M38G replication following low dose rQ-M38G infection (MOI = 0.001, 1 virus per 1000 cells).

We assessed transgene expression, virus replication and cytotoxicity following rQ-M38G infection of 5 human tumor cell lines. SK-NEP_Luc (Ewing sarcoma) demonstrated elevated GLuc expression, virus replication and cytotoxic susceptibility similar to Vero cells. Osteomet (Osteosarcoma), STS26T_dsRed (MPNST), and S462.TY (MPNST) each demonstrated lower sensitivity in all three assays. Finally, we assessed cytotoxicity in 3 well established mouse tumor models derived from a C57/Bl6 background (Fig. S3) and several spontaneous murine thyroid or small cell lung tumor lines generated by a collaborator (data not shown). HGF116 (rhabdomyosarcoma) was the only cell line showing any measurable cytotoxicity, but only at a high virus dose (MOI = 1, 1 infectious virus per cell). These data identified SK-NEP_Luc as a prime target for *in vivo* screening using rQ-M38G while other human tumor cell lines were predicted to be less amenable to screening with rQ-M38G.

### Systemic administration of rQ-M38G to identify tumor presence

**Figure 3:**
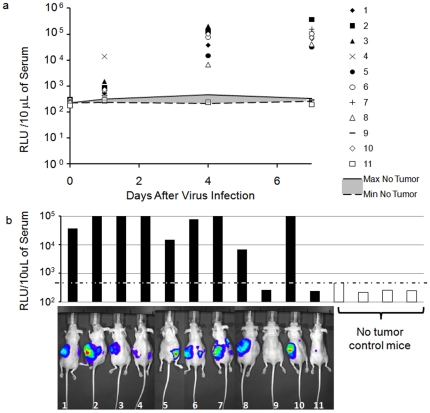
Detection of tumor presence by serum biomarker levels. (**a**) Time course of GLuc expression in renal subcapsule (r.s.c) SK-NEP_Luc-bearing and tumor-free mice injected systemically with 1.2×10^7^ pfu or rQ-M38G. Numbers in legend represent individual mice. (**b**) GLuc expression and *in vivo* imaging of SK-NEP_Luc-bearing mice four days following systemic injection of 1.2×10^7^ pfu of rQ-M38G. Serum GLuc levels for mice injected with tumor and control that did not receive tumor injections (bar graph), and *in vivo* luciferase imaging of mice that received tumor cell injections.

We tested the suitability of rQ-M38G as a gene delivery vector to force tumor-specific secretion of a biomarker following systemic administration of rQ-M38G in mice with and without tumors. Eleven mice were injected orthotopically with 10^6^ SK-NEP_Luc cells into their renal subcapsule. Tumor-implanted mice and control tumor-free mice were subsequently injected intravenously (i.v) with 1.2×10^7^ pfu of rQ-M38G 5 weeks after tumor implantation. On days 1, 4 and 7 following virus injection mice were imaged to identify firefly luciferase-positive tumors and blood samples were collected by retro-orbital eye bleed and assayed for serum GLuc (Fig. 3a). *In vivo* imaging identified 10 out of 11 mice which received tumor cell injections had formed tumors (Fig. 3b). One mouse (#9) never formed a tumor and one mouse (#11) had a tumor that was scarcely detectable. In all mice where tumor burden was apparent (9 out of 11), serum GLuc levels were 15- to 440-fold higher than tumor-free mice, whereas serum GLuc from the other two mice remained below control mouse serum GLuc levels (Fig. 3). Therefore, rQ-M38G administered systemically to induce biomarker production resulted in a detection sensitivity of 90% for SK-NEP_Luc-bearing mice. Similar results were for tumors in different anatomical sites (Fig. S4).

**Figure 4:**
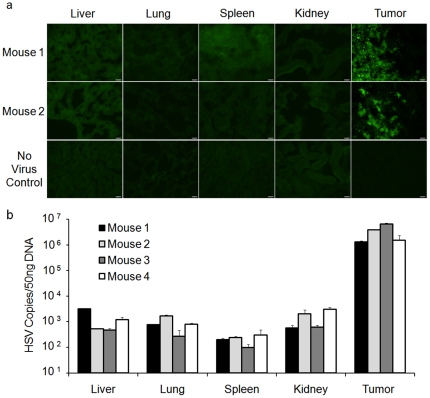
Virus biodistribution. (**a**) GFP immunofluoresence in SK-NEP_Luc-bearing mice with and without systemic virus administration. Mice receiving virus (#1 and #2) showed selective GFP expression in tumor, while mice receiving no virus (No Virus Control) showed global GFP-negativity. Scale bar = 15 microns. (**b**) Biodistribution of virus in mice with SK-NEP_Luc tumors following systemic administration as determined by qPCR for viral genomes.

To determine the location of rQ-M38G in tissues following i.v. injection and confirm that the serum GLuc signal was being expressed from tumors and not normal organs, organs were harvested from mice 7 days after virus infection and subjected to immunofluorescence for GFP expression and quantitative PCR for virus genomes. Only tumors demonstrated GFP expression in both control (data not shown) and experimental mice (Fig. 4a) injected systemically with rQ-M38G. Quantitative PCR for virus genome copies reflected the same distribution of virus in tumor-bearing mice as was seen with GFP expression where virus copies were at least 2100–fold higher in the tumors than healthy tissues (Fig. 4b). Immunofluorescence and qPCR indicate that virus infection predominated in the tumor while being minimal in healthy tissues. Therefore, systemically administered rQ-M38G successfully identified the presence of SK-NEP_Luc tumors by forcing tumors to produce a secretable biomarker. We demonstrated similar findings in tumor models that are less susceptible to virus infection including models of malignant peripheral nerve sheath tumors (in both subcutaneous and intraperitoneal locations) and osteosarcoma (Figures S5, S6, S7).

### Induction of GLuc expression in tumors by intratumoral virus injection

A clear difference was observed between SK-NEP_Luc and the three other tumor models tested which each showed lower *in vitro* cytotoxicity and GLuc expression coupled with lower biomarker expression *in vivo*. To identify whether delivering larger doses of virus to tumors could increase *in vivo* biomarker expression, we administered rQ-M38G directly into tumors by intratumoral injection in models of S462.TY and Osteomet.

Mice bearing subcutaneous flank Osteomet tumors greater than 200 mm^3^ were injected with 1.9×10^5^ pfu of virus. Blood samples were collected and analyzed for GLuc at each time point (Fig. S7a) while two mice were sacrificed at each time point and analyzed for viral genomic copies by qPCR (Fig. S7b). Serum GLuc levels in blood from mice bearing tumors injected with rQ-M38G was higher than GLuc levels in control uninfected mice, and increasing GLuc over time traced a profile similar to viral copy number in tumors. Despite a 4000-fold genomic amplification in tumors, serum GLuc levels rose only 10-fold above uninfected controls. Intratumoral injection with 8×10^3^ pfu or 8×10^7^ pfu of virus was repeated in S462.TY tumors when tumors were larger than 500 mm^3^. Four days after i.t. injection serum GLuc levels were assayed as plotted in Fig. S8. All mice receiving intratumoral injection of virus demonstrated higher GLuc levels than tumor-free controls without a significant difference in GLuc concentration between the low dose and 10,000-fold high virus dose, suggesting saturation at the low dose (at least via the intratumoral route).

### Biomarker sensitivity

Small animal models used to detect minimal tumor burdens present theoretical scalability challenges when translating findings to human-scale practicality. To address these challenges we sought to evaluate theoretical detection limits for our biomarker based on an *in vitro* assay and detect small tumor burdens *in vivo* by 3 methods: IVIS luminescence imaging, virus-mediated GLuc expression in serum and post-mortem histological analysis. The blood volume in a mouse is approximately 2 mL (8% of the 25 g total body weight [30]). Spherical SKNEP-Luc tumor comprised of 10^6^ cells was determined to be 1.83 mm in diameter by measuring volume occupied in a centrifuged sample of an equal number of cells, assuming small tumors are comprised predominantly of tumor cells. Ten to 1 million cells were plated in 2 mL of cell culture media and co-infected with rQ-M38G at an MOI of 3 to ensure every cell would be infected. Two days after infection cell culture media was collected and analyzed for GLuc concentration. Virus-mediated GLuc expression could be reliably resolved by simultaneously transducing 1,000 cells, which approximates a spherical tumor of diameter 183 microns (Fig. S9).

**Figure 5:**
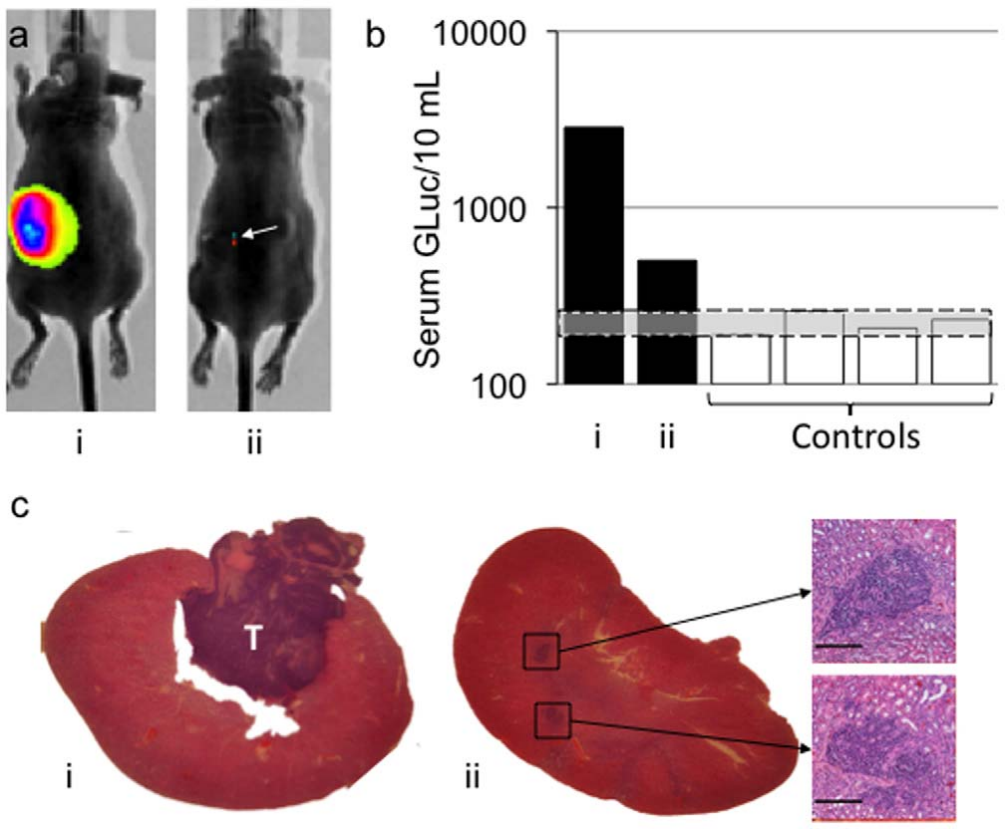
Sensitivity limits of detection. a) *in vivo* imaging of two mice (*i* and *ii*) with vastly different SKNEP-Luc tumor burdens. b) Serum GLuc concentration in mouse i, ii and four tumor-free control mice 4 days following systemic infection with 1×10^7^ pfu of rQ-M38G. c) Hematoxylin and eosin macroscopic images of tumor bearing kidneys (T = tumor, scale bars = 1 mm) and insets of microscopic tumor foci in the renal parenchyma of mouse *ii* (scale bar = 200 µm).

Ewings sarcoma tumors (SKNEP-Luc) of different sizes were established in 11 mice by injecting 10^6^ cells into the left kidney subcapsularly in 3 groups of mice on 3 consecutive weeks. Mice with tumors that had developed over 2, 3 and 4 weeks and 4 tumor-free control mice were infected systemically with 1×10^7^ pfu of rQ-M38G. Four days following infection mice were imaged via IVIS, blood samples collected for GLuc quantitation, and mice were sacrificed for histological tumor sizing. *In vivo* imaging for luciferase expression revealed mice baring tumors drastically different in size at the time of infection and sacrifice (extreme examples shown in Fig. 5a). Serum GLuc concentration in tumor bearing mice 4 days after infection revealed GLuc levels greater than similarly infected tumor-free control mice (Fig. 5b). Histological evaluation of tumors in each mouse revealed a macroscopic tumor and microscopic tumor foci in mouse *i* and *ii* respectively (Fig. 5c).

## Discussion

We developed a novel cancer screening strategy whereby tumors are forced to secrete a biomarker. We developed a conditionally replicative HSV, rQ-M38G, to selectively force tumor cells to secrete Gaussia luciferase as a demonstration of this screening strategy. Tumor-bearing animals given intravenous rQ-M38G expressed higher levels of biomarker compared with tumor-free animals, which showed only low, background expression. The utility of rQM-38G for screening appeared to be a function of the capability of a given tumor to support HSV replication. Regardless of tumor type or location, tumor screening by rQ-M38G was highly sensitive to tumor presence.

A critical feature for cancer screening is the ability to detect small tumors, ultimately in people. One *a priori* concern was that small tumors may be associated with less vascular surface area available for HSV entry. This issue did not appear to be a limitation in mice, because in some of the models we were able to detect tumors as small as 4–5 mm in diameter (50 mm^3^) and in another model we detected microscopic tumor burden. Scalability to humans (with larger blood volumes) is difficult to predict, but some estimates are possible. *In vitro* evaluation of increasing numbers of SKNEP-Luc tumor cells indicates that infecting as few as 1,000 cells in a volume of cell culture media equal to a mouse blood volume yields detectable GLuc secretion. Therefore, simultaneous expression of GLuc from 1,000 cells at any time in a mouse is theoretically detectable. Under these conditions infecting 10% of the cells in a tumor of diameter 400 µm (10^4^ cells) would be detectable in a mouse. When scaled from a mouse to a human the theoretical limit of detection is increased by a ratio of 1∶2600 (25 mg mouse∶65 kg human) and diluting the biomarker in a larger average human blood volume of 4.7 L. Using these numbers, the theoretical limit of detection when infecting 10% of the cells in a tumor is changed from a 394 um diameter mass in a mouse to a 1–4 mm tumor diameter in a human. If only 1% of tumor cells are transduced by virus, tumors as small as 8.5 mm in diameter might still be detectable using these calculations. This sensitivity suggests strong potential for identifying minimal tumor burdens even when scaled to human proportions.

The ability of this screening strategy to reveal tumors is dependent upon viral factors, tumor microenvironment, and biomarker detection limitations each of which can be enhanced for real world practicality. Here we employed a doubly attenuated HSV to effect a tumor specific transduction and gene expression. Such vectors have already been documented to be safe for human use [31]. Alternative viruses with single or less-attenuating mutations demonstrate greater oncolytic capacity are also already in clinical trials (e.g., HSV1716, see www.clinicaltrials.gov, NCT00931931). Coupling this screening strategy to less attenuated viruses will likely diversify its utility among the various solid malignancies as well as enhance the therapeutic component. Agents that are simultaneously both diagnostic and therapeutic have been dubbed “theragnostic.” It is possible our strategy could be further refined by the use of a more robust cancer-dependent promoter to drive biomarker expression. Improved vector delivery to the tumor may also be achieved using tumor targeting small molecule and nanoparticles uptake-enhancement strategies as recently described with the use of internalizing RGD peptides [8]. Eventual commercialization of our proposed screening strategy would benefit from using biomarkers that are already in widespread use such as βHCG (pregnancy test), PSA (prostate cancer) and αFP (liver and germ cell malignancies). Biomarker assay sensitivity in the context of this screening strategy can still improve exponentially as is being realized with microfluidic technologies [32], [33], [34], [35].

A significant concern for the prototypical cancer screening method developed in this work is the development of an immune response to the gene delivery agent or the transduced biomarker that would preclude repeated use of the screening tool. Previous work in the field has shown that pre-immunized animals still experience therapeutic benefit from HSV with sustained efficacy [36]. Because 80% of the general population has been exposed to HSV, implementing this detection strategy with HSV will depend upon the efficacy of engineered HSVs administered to immunocompetent subjects who have previously been exposed to wild-type HSV strains. *In vitro* screening for mouse tumor cell lines revealed that all mouse lines we tested exhibited comparably low susceptibility to infection by our attenuated mutant virus, on par with normal quiescent human cells. In vivo models of HGF116 demonstrated no virus sensitivity following i.t. or i.v. injection. Thus, we are unable to assess our cancer screening strategy in an immunocompetent model until the identification of mouse models that are more susceptible to human HSV. Implementation of this strategy may evolve with non-viral vectors [37] or viral vectors masked in liposomes [38].

The challenge of population-wide cancer screening is the development of clinical assays that are fiscally practical, universal across a spectrum of cancer types, and easy to implement. Physical examination, while affordable, often fails to identify malignancies that are deep or asymptomatic. Imaging benefits from high sensitivity, but may not always differentiate nonspecific or benign masses from malignant disease (eg, lung nodules may be granuloma or cancer), is financially challenging and difficult to broadly implement. With the identification of appropriate biomarkers, cancer screening could potentially be economically viable with automated point of care testing (POCT) technologies (www.i-stat.com, www.biosite.com, www.siloambio.com [39]).

This work demonstrates the principle of inducing expression of a secretable transgene in cancer using a systemically administered gene delivery agent as a biomarker to screen for tumor presence. The novel screening principle proposed and demonstrated in this work could hold immediate implications for patients with known cancer risks such as genetic predispositions (BRCA-1, Nf1 mutations) or patients with a history of cancer who are at high risk for recurrence. Ultimately, exogenous administration of a cancer targeting gene delivery agent (infectious or not) could force any malignancy to secrete a biomarker and could be used as a universal first step for population-wide cancer screening. The impact of this approach would revolutionize the technology for cancer detection in industrialized nations and the developing world where imaging and biopsy-based diagnostics may not be as readily available.

## Materials and Methods

### Cells and Viruses

Human tumor cell lines have been described previously [40], [41] or were purchased from American Type Culture Collection (ATCC, Manassas, VA) except SK-NEP_Luc, which was a kind gift from Jason Frischer (CCHMC, Cincinnati, OH). Cells were grown in DMEM or McCoys 5A medium (ATCC, SK-OV-3, SK-NEP_Luc) with 10% fetal bovine serum (Hyclone, Logan, UT) and penicillin/streptomycin (Life Technologies, Carlsbad, CA). Mouse C57/Bl6 cell lines LLC^GFP^ [42] (Lewis lung carcinoma) and B16-BL6 (melanoma) were kind gifts from Joe Palumbo (CCHMC, Cincinnati, OH) and HGF116 (rhabdomyosarcoma) was derived from a genetically engineered mouse. Primary human foreskin keratinocytes (HFK) were a kind gift from Susa Wells (CCHMC, Cincinnati, OH), grown in EpiLife Media (Cascade Biologics, Portland, OR) and differentiated into quiescent keratinocytes with 10% FBS and 1 mmol/L CaCl_2_ [43].

rQ-M38G was constructed as follows: The U_L_38 promotor was isolated from HSV rRp450 by PCR using primers designed previously [18] that were modified to include a 5′ BglII and 3′ HindIII sites (F1, R1 in Table S1) and cloned into the BglII and HindIII sites of pCMV-GLuc (Invitrogen), replacing the CMV promoter. The U_L_38p-GLuc cassette was amplified by PCR from pU_L_38p-GLuc with inclusion of a 5′SpeI and 3′ EcoRI and cloned into the SpeI-EcoRI sites of the “HSV-Quick” shuttle plasmid, pT-OriSIE4/5 [29] (a kind gift from Yoshinaga Saeki, The Ohio State University, Columbus, Ohio), in which the oriS(E4/5 Kpn I fragment had been removed. The resulting plasmid, pT-U_L_38p-GLuc, was used in the HSVQuick BAC system to generate rQ-M38G [29]. Primers for PCR analysis and sequencing are shown in Table S2. Viruses were propagated and titered [44] by plaque assay on Vero cells. Cell cytotoxicity was determined in 96-well tissue culture plates using a modified MTS/PMS assay (Promega, Madison, WI).

### Gaussia luciferase assay

GLuc activity was assessed on 10 µL samples of tissue culture media or serum by chemiluminescent assay [45] on an autoinjecting luminometer by injecting 50 µL of 50 µM coelenterazine (Prolume, Pinetop, AZ) to each sample in triplicate and integrating the signal for 2.5 seconds [26].

### Virus biodistribution

Animal studies were approved by the Cincinnati Children's Hospital Institutional Animal Care and Use Committee (Animal protocol #9D12095). 5–6-week old female Balb/c athymic nude mice (Harlan Sprague Dawley, Indianapolis, IN) were injected with 1–5 million cells. Cells were prepared in 33% Matrigel (Becton Dickinson, Bedford, MA) and 66% phosphate buffered saline (PBS, pH 7.4) for subcutaneous (s.q.) injection and PBS only for renal subcapsule (r.s.c) or intraperitoneal (i.p.) injection. Virus doses are described in the text. Virus was suspended in 100–150 µL of PBS for tail vein injections and 50–100 µL of PBS for intratumoral injections, which were distributed into 5 fractions throughout the tumor.

### Tissue preparation and immunofluorescence

Tissues were fixed, embedded, and cut into 12-micron sections using standard procedures. Sections were permeablized with 0.2% Triton-X in PBS for 10 minutes, blocked in 10% normal goat serum for 60–90 minutes, and incubated with Chicken anti-GFP (#16901, Millipore/Chemicon, Billerica, MA) for 60 minutes, rinsed three times with PBS, and incubated with secondary antibody (Goat anti-Chicken FITC, Jackson ImmunoResearch, West Grove, PA), rinsed with PBS, and mounted with Fluoromount-G (Electron Microscopy Sciences, Hatfield, PA). All slides were then imaged with OpenLab Imaging Software (Improvision, Waltham, MA) on an inverted fluorescent Zeiss microscope.

### HSV genome quantitation

DNA was isolated from mouse tissues using the Gentra Puregene DNA isolation kit (Qiagen, Valencia, CA) and quantified using the NanoDrop 2000 spectrophotometer (Thermo Scientific, Wilmington, DE). Real-time PCR was performed using the ABI 7500 system (Applied Biosystem, Foster City, CA) [46]. Standards were made from purified HSV1716 viral DNA.

### rQ-M38G gene expression and replication studies in cell lines

Cells were infected in 12-well dishes for 1 hour and harvested at times indicated. 100 uL of the cell suspension was used for measuring Gaussia luciferase, while the remaining cell suspension was subjected to three cycles of freeze–thaw and centrifuged at 20,000× g. Pellets were resuspended in 200 uL PBS. DNA was isolated using the DNeasy Blood and Tissue kit (Qiagen, Valencia, CA). Fast real-time PCR was performed using the 7900HT Fast Real Time PCR System (Applied Biosystem, Foster City, CA). Standard DNA or DNA extracted from infected cells (40 ng) was added to 10 µL of SYBR Premix Ex Taq II Kit (TaKaRa, Otsu, Shiga, Japan), 1.6 µL of a thymidine kinase primer mixture (TK 290-F: 5′ TCG CGA ACA TCT ACA CCA CAC AAC; TK 400-R: 5′ CGG CAT AAG GCA TGC CCA TTG TTA; each at 400 nM), 0.4 µL Rox (TaKaRa) and PCR-grade water to a final volume of 20 µl per reaction. PCR was 1 cycle of 95°C for 30 seconds, 40 cycles of 95°C for 3 seconds, 60°C for 15 seconds, and 72°C for 25 seconds.

### *In vivo* imaging

Mice were injected i.p. with 150 mg/kg of D-luciferin (Caliper Life Sciences, Hopkinton, MA) and imaged by IVIS200 (Calipur Lifesciences).

## Supporting Information

Figure S1 GLuc excretion following infection of Vero cells and 4 human tumor cell lines (100,000 cells/well) with rQ-M38G across a range of MOIs.
(TIF)

Figure S2 Virus replication as measured by qPCR following infection of Vero cells and 4 human tumor cell lines (100,000 cells/well) with rQ-M38G across a range of MOIs.
(TIF)

Figure S3 In vitro cytotoxicity of rQ-M38G in a well characterized virus permissive cell line (Vero), replicating and non-replicating human keratinocytes (HFK-r and HFK-q respectively), 5 human tumor cell lines (S462.TY, Osteomet, SK-OV-3, STS26T_dsRed, and SK-NEP_Luc) and 3 mouse tumor cell lines (LLC^GFP^, B16-Bl6, HGF116).
(TIF)

Figure S4 Serum GLuc levels following i.v. infection with 1×10^7^ pfu of rQ-M38G in mice bearing intraperitoneal, intramuscular and subcutaneous tumors.
(TIF)

Figure S5 GLuc expression in two MPNST tumor models (S462.TY and STS26T_dsRed) with low *in vitro* virus sensitivity and GFP expression in S462.TY-bearing mice. (**a**) Serum GLuc following systemic injection of 5×10^7^ pfu of rQ-M38G by tail vein into mice bearing S462.TY subcutaneous tumors. Shaded gray represent the range of GLuc levels for tumor-free mice which received the same dose of virus; (**b**) Serum GLuc levels following systemic administration of 1×10^7^ pfu of rQ-M38G in mice bearing subcutaneous STS26T_dsRed tumors; and (**c**) GFP expression in 2 S462.TY-bearing mice (#*iii* and #*vii*) demonstrating few punctate GFP positive cells in tumors and no GFP positive cells in healthy tissues. Tumor volume (mm^3^) at time of virus injection is noted for each mouse in the plot legends.
(TIF)

Figure S6 Serum GLuc levels following systemic administration of 1×10^7^ pfu of rQ-M38G in mice which were injected with 2×10^6^ STS26T_dsRed cells intraperitoneally.
(TIF)

Figure S7 Serum GLuc levels (a), and number of virus copies in tumors as determined by qPCR (b) following direct intratumoral injection of rQ-M38G into subcutaneous Osteomet tumors larger than 200 mm^3^. c) Serum GLuc levels in a subcutaneous *in vivo* model for Osteomet following systemic injection of 1.9×10^7^ pfu or 1.9×10^5^ pfu of rQ-M38G. Gray shaded area represents serum GLuc levels in tumor free mice also receiving 1.9×10^7^ pfu of rQ-M38G. Key identifies mouse number, virus dose, and tumor size at time of injection respectively.
(TIF)

Figure S8 Serum GLuc levels from S462.TY bearing mice four days following i.t. injection of 8×10^3^ pfu or 8×10^7^ pfu of rQ-M38G.
(TIF)

Figure S9 In vitro modeling of increasing numbers of cells (SKNEP-Luc), their theoretical tumor volume and GLuc production from infection of every cell.
(TIF)

Table S1 Primers used in the production of HSV-UL38p-GLuc (rQ-M38G).
(TIFF)

Table S2 U_L_38-GLuc sequence.
(TIFF)
