# Supplementary figures and images for: Cancer Screening by Systemic Administration of a Gene Delivery Vector Encoding Tumor-Selective Secretable Biomarker Expression
Source: PLoS One. 2011 May 11;6(5):e19530. doi: 10.1371/journal.pone.0019530 (PMC3092745; doi:10.1371/journal.pone.0019530)

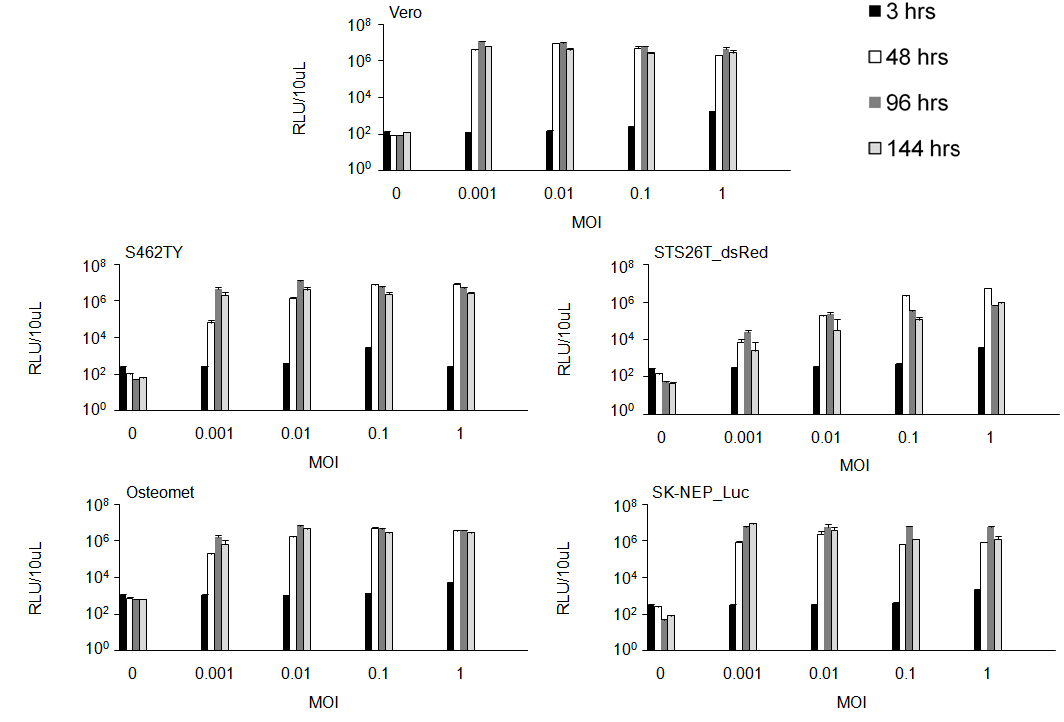

Supplement: Figure S1 — GLuc excretion following infection of Vero cells and 4 human tumor cell lines (100,000 cells/well) with rQ-M38G across a range of MOIs. (TIF) [file pone.0019530.s001.tif]

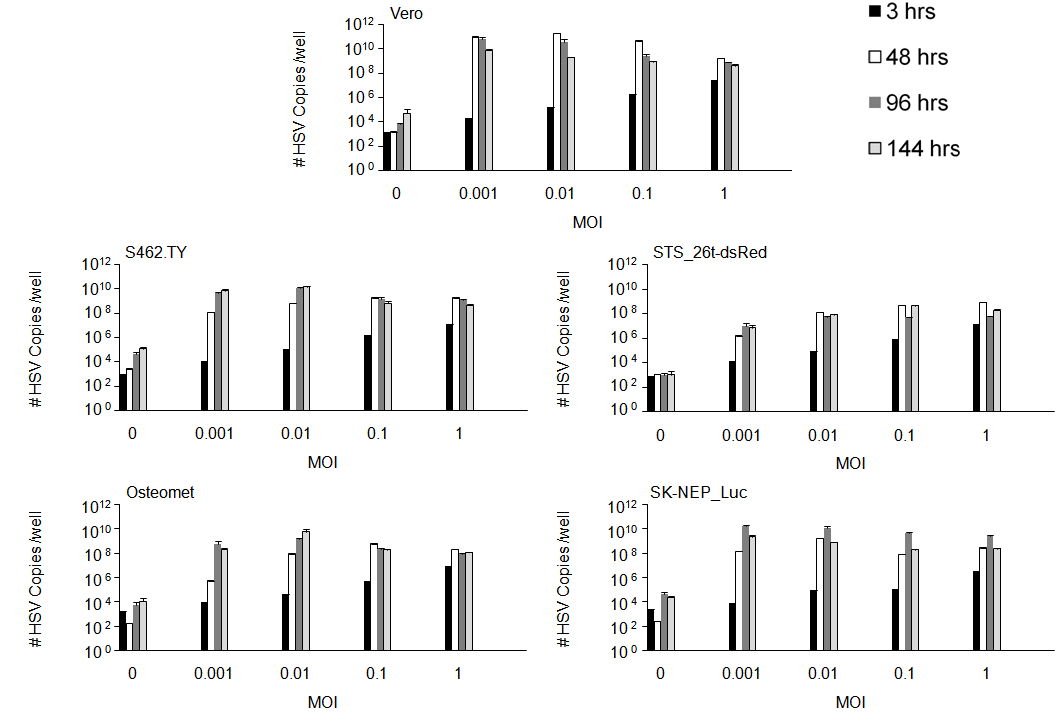

Supplement: Figure S2 — Virus replication as measured by qPCR following infection of Vero cells and 4 human tumor cell lines (100,000 cells/well) with rQ-M38G across a range of MOIs. (TIF) [file pone.0019530.s002.tif]

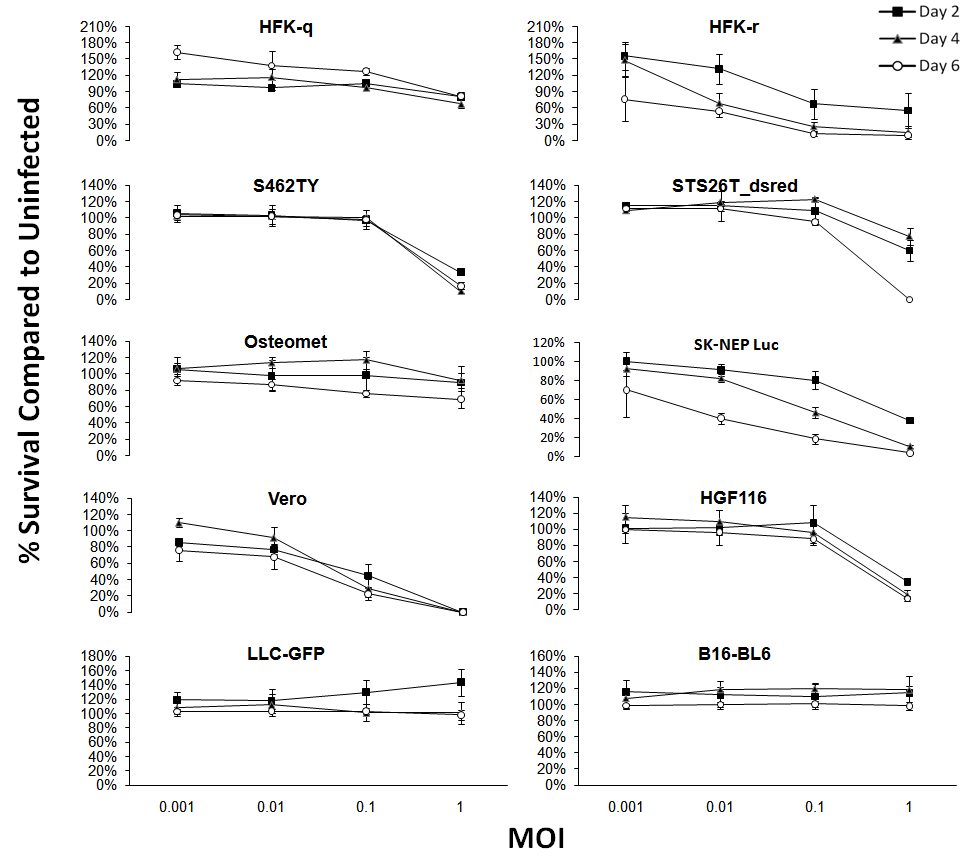

Supplement: Figure S3 — In vitro cytotoxicity of rQ-M38G in a well characterized virus permissive cell line (Vero), replicating and non-replicating human keratinocytes (HFK-r and HFK-q respectively), 5 human tumor cell lines (S462.TY, Osteomet, SK-OV-3, STS26T_dsRed, and SK-NEP_Luc) and 3 mouse tumor cell lines (LLCGFP, B16-Bl6, HGF116). (TIF) [file pone.0019530.s003.tif]

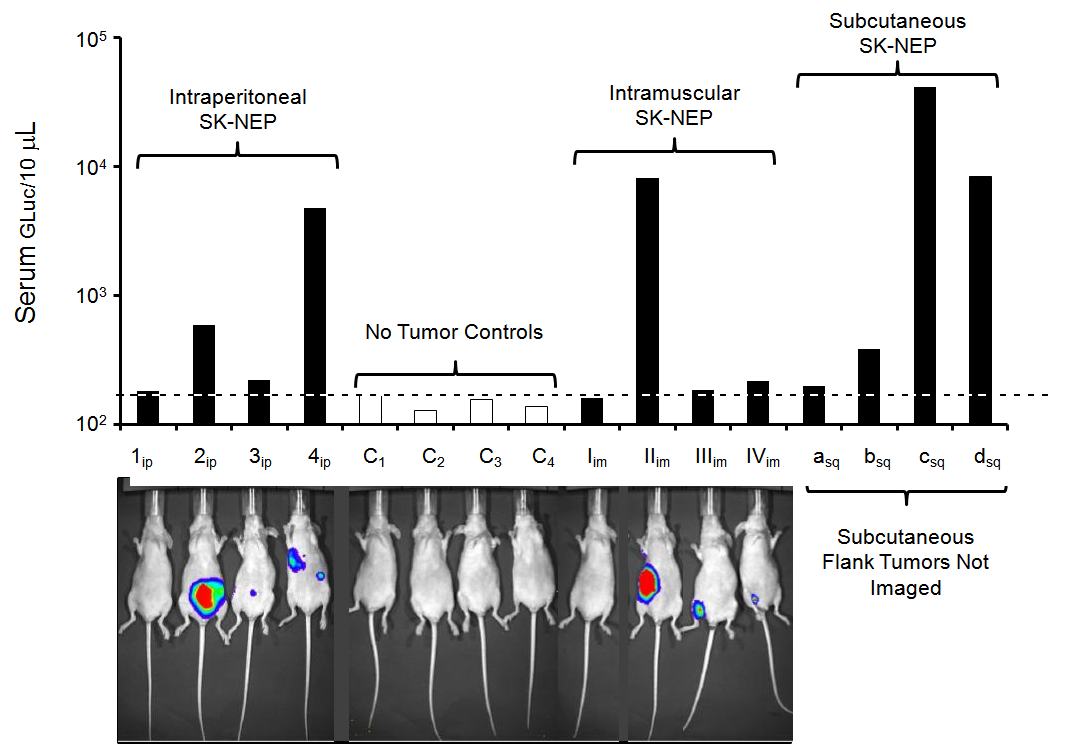

Supplement: Figure S4 — Serum GLuc levels following i.v. infection with 1×10^7^ pfu of rQ-M38G in mice bearing intraperitoneal, intramuscular and subcutaneous tumors. (TIF) [file pone.0019530.s004.tif]

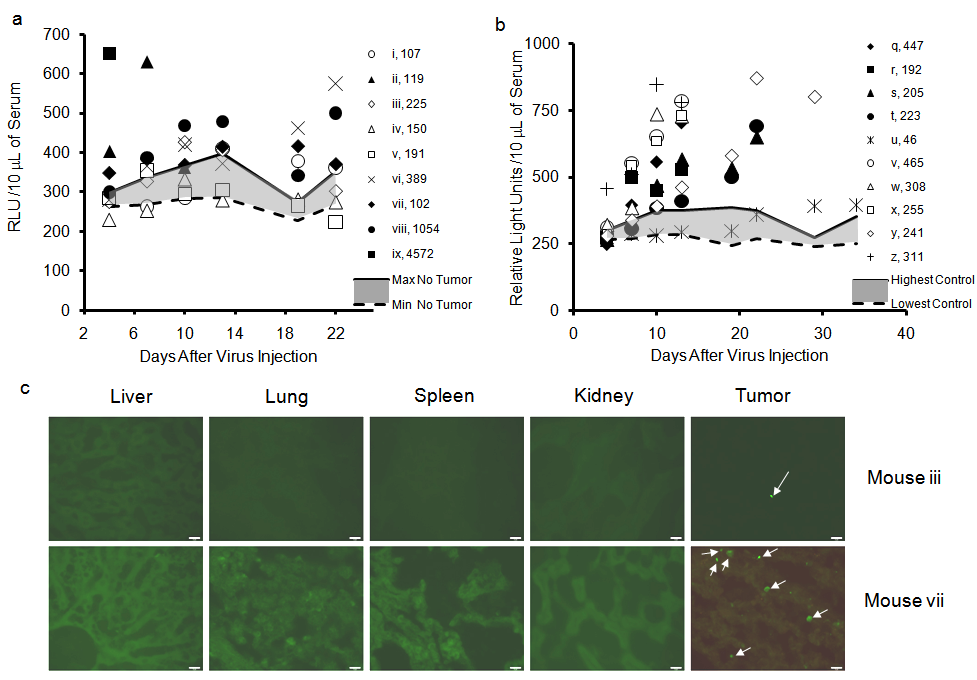

Supplement: Figure S5 — GLuc expression in two MPNST tumor models (S462.TY and STS26T_dsRed) with low in vitro virus sensitivity and GFP expression in S462.TY-bearing mice. (a) Serum GLuc following systemic injection of 5×10^7^ pfu of rQ-M38G by tail vein into mice bearing S462.TY subcutaneous tumors. Shaded gray represent the range of GLuc levels for tumor-free mice which received the same dose of virus; (b) Serum GLuc levels following systemic administration of 1×10^7^ pfu of rQ-M38G in mice bearing subcutaneous STS26T_dsRed tumors; and (c) GFP expression in 2 S462.TY-bearing mice (#iii and #vii) demonstrating few punctate GFP positive cells in tumors and no GFP positive cells in healthy tissues. Tumor volume (mm3) at time of virus injection is noted for each mouse in the plot legends. (TIF) [file pone.0019530.s005.tif]

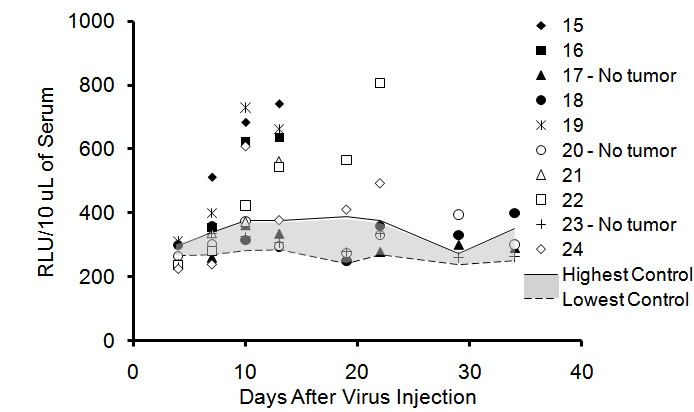

Supplement: Figure S6 — Serum GLuc levels following systemic administration of 1×10^7^ pfu of rQ-M38G in mice which were injected with 2×10^6^ STS26T_dsRed cells intraperitoneally. (TIF) [file pone.0019530.s006.tif]

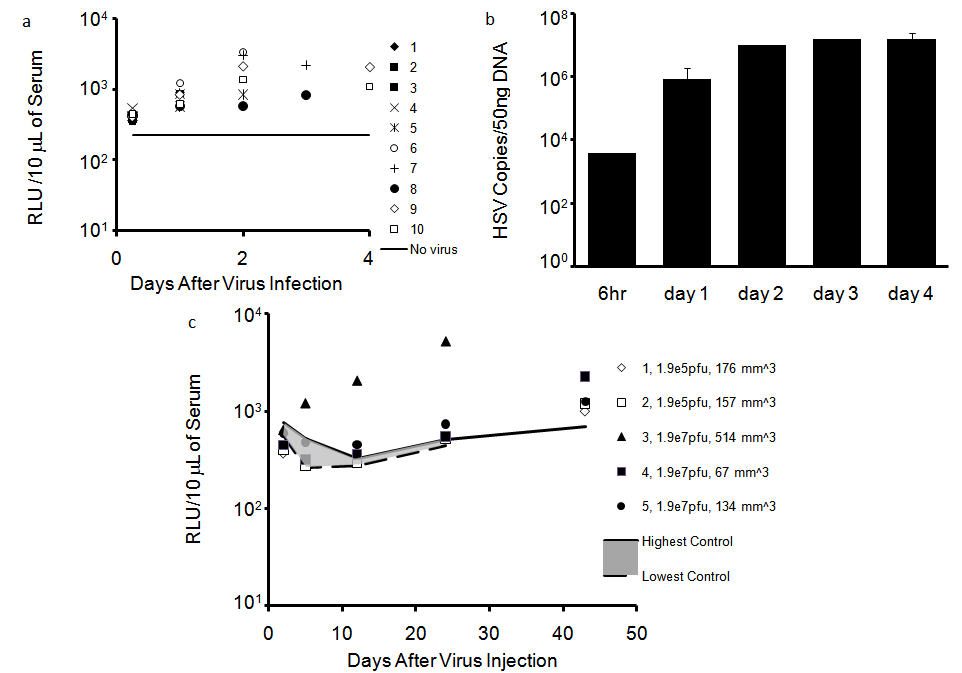

Supplement: Figure S7 — Serum GLuc levels (a), and number of virus copies in tumors as determined by qPCR (b) following direct intratumoral injection of rQ-M38G into subcutaneous Osteomet tumors larger than 200 mm3. c) Serum GLuc levels in a subcutaneous in vivo model for Osteomet following systemic injection of 1.9×10^7^ pfu or 1.9×10^5^ pfu of rQ-M38G. Gray shaded area represents serum GLuc levels in tumor free mice also receiving 1.9×10^7^ pfu of rQ-M38G. Key identifies mouse number, virus dose, and tumor size at time of injection respectively. (TIF) [file pone.0019530.s007.tif]

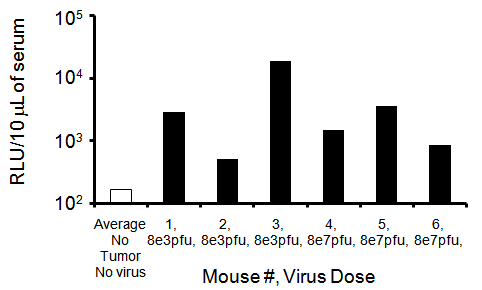

Supplement: Figure S8 — Serum GLuc levels from S462.TY bearing mice four days following i.t. injection of 8×10^3^ pfu or 8×10^7^ pfu of rQ-M38G. (TIF) [file pone.0019530.s008.tif]

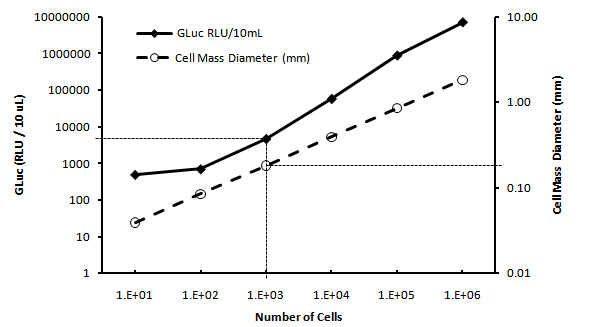

Supplement: Figure S9 — In vitro modeling of increasing numbers of cells (SKNEP-Luc), their theoretical tumor volume and GLuc production from infection of every cell. (TIF) [file pone.0019530.s009.tif]

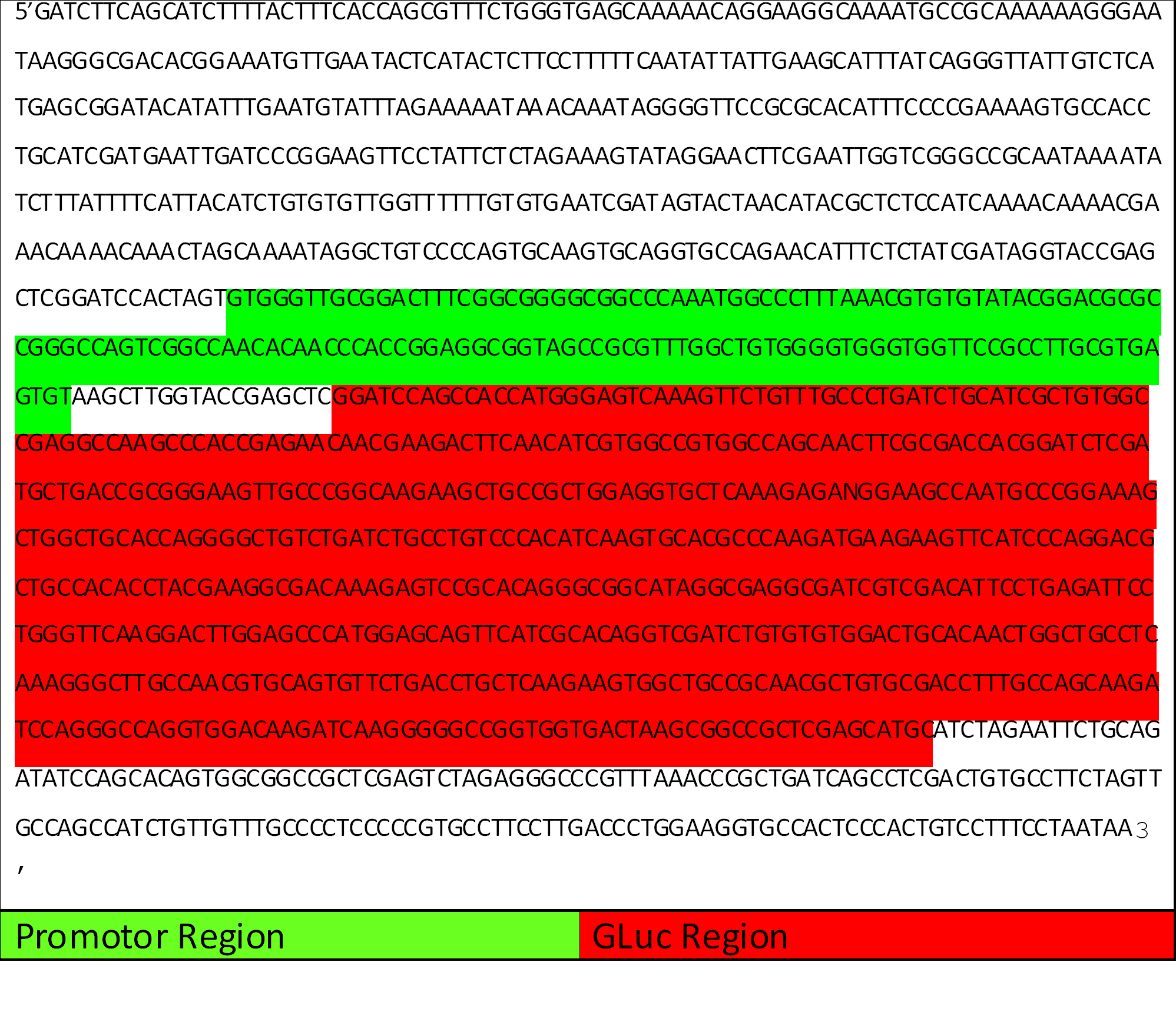

Supplement: Table S1 — Primers used in the production of HSV-UL38p-GLuc (rQ-M38G). (TIFF) [file pone.0019530.s010.tiff]
